# The Development of a Digital Twin Framework for an Industrial Robotic Drilling Process

**DOI:** 10.3390/s22197232

**Published:** 2022-09-23

**Authors:** Ahmad Farhadi, Stephen K. H. Lee, Eoin P. Hinchy, Noel P. O’Dowd, Conor T. McCarthy

**Affiliations:** 1 Confirm Centre, University of Limerick, V94 C928 Limerick, Ireland; 2 Bernal Institute, University of Limerick, V94 T9PX Limerick, Ireland; 3 School of Engineering, University of Limerick, V94 T9PX Limerick, Ireland

**Keywords:** smart manufacturing, robotic machining, digital twin

## Abstract

A digital twin is a digital representation of a physical entity that is updated in real-time by transfer of data between physical and digital (virtual) entities. In this manuscript we aim to introduce a digital twin framework for robotic drilling. Initially, a generic reference model is proposed to highlight elements of the digital twin relevant to robotic drilling. Then, a precise reference digital twin architecture model is developed, based on available standards and technologies. Finally, real-time visualisation of drilling process parameters is demonstrated as an initial step towards implementing a digital twin of a robotic drilling process.

## 1. Introduction

The demanding requirements for aerospace components including engine parts or body structure necessitate a reliable manufacturing process. Therefore, continuous monitoring, controlling and optimisation of equipment performance, product development and manufacturing is essential. Machining is an important manufacturing process in aerospace part fabrication, encompassing different physical phenomena such as fracture, deformation, heat dissipation, tribology and vibration [1]. Among machining processes, drilling is used primarily in the final production stage of aircraft structures (e.g., fuselage, wings, etc.), with robotic arms for precise and cost-efficient drilling operations [2]. With the rapid development of manufacturing industry through digital transformation, a digital twin is a new technology with the potential to improve machining efficiency and reducing imperfections relating to robotic drilling.

Digital twin is a concept in Industry 4.0 that provides a digital representation of a manufacturing element through a stream of data collected from the physical world. These data are used to update digital entities, and control commands are sent back to manufacturing elements for manipulation. Therefore, a seamless, continuous information exchange between physical and digital twins occurs allowing prediction and optimisation of manufacturing processes [3]. In the manufacturing environment, the digital twin implementation is quite diverse depending on the desired objectives. In product design and development, digital twin can also play an important role in data structures and engineering processes for digitalization and constructing digital thread [4]. In manufacturing systems, the digital twin is used to improve service management throughout product life cycle including design, manufacturing, transportation and after sale service [5].

Although the definition and concept idea of the digital twin is now established, building a unified digital twin framework for manufacturing environment is missing. Many researchers have proposed platforms to construct a digital twin for a specific discipline but, none is comprehensive and inclusive. Therefore, this work introduces a framework to develop a digital twin for robotic drilling based on ISO 23247-Digital twin framework for manufacturing [6]. Section 2 provides an overview of robotic machining, its research areas, and implementations. Section 3 gives a review of the history of digital twin from conceptual idea to building frameworks, and supports the objective of this research. In Section 4, a digital twin architecture for robotic drilling is presented. Finally, Section 5 demonstrates a use case of digital twin for robotic drilling. 

## 2. Robotic Drilling

Computer numerical control (CNC) machine tools are currently the main systems used to perform machining operations. However, industrial robots have considerably more dexterity than CNC machines and are more cost efficient and flexible [7,8]. Robot controllers and motion planners are used to manage robot motion, control particulate trajectories and movement speeds during drilling. Most controllers can integrate end-effectors such as cameras and sensors to monitor the process [9]. To machine large parts, linear axes are typically used as a 7th axis. Automated guided vehicles have also been employed for robotic machining of large components [10]. Some examples of robotic machining include polishing [11], grinding [12], milling [13], drilling and riveting [14] and machining of large structures [15]. A robotic drilling system typically consists of a six-axis industrial robot equipped with a drilling end-effector and a system to control robot movement and drilling process execution [16]. To improve the efficiency of the joining process, robots can be fitted with multifunctional end-effectors designed to perform a sequence of process steps including clamping, drilling and riveting without the need for robot movement.

Although robotic drilling offers increased flexibility and speed, hole quality and drilling performance may be compromised due to errors during the process [17]. To increase machining performance and hole quality, different methods are implemented to compensate robot trajectory errors, drilling nose slippage on the workpiece surface and drilling axis adjustment. It has been shown that positioning and perpendicularity can be improved by using multi sensor measurement systems [14] and external sensor-based and compliance model-based compensation strategies [18]. Implementing closed-loop force-controlled feedback is another approach to improve hole positioning and quality [19]. From the research presented, it is evident that robotic drilling needs further investigation, regarding error compensation and process accuracy through online monitoring and real time optimization of deficiencies during the drilling process. A digital twin has the potential for observation of the drilling process and predicting and mitigating errors during robotic drilling and is the topic of this work. 

## 3. The History of the Digital Twin from Concept Idea to Building Frameworks

A Product Lifecycle Management (PLM) model incorporating a “digital twin” was proposed in 2003 [20]. In the proposed model, conceptual ideal for PLM was referred to as the Mirrored Spaces Model. This model had the fundamental elements of a digital twin, which are real space, virtual space and flow of data between them. Later, the components of a digital twin were categorized into “*prototype, instance and aggregate*” [21]. The term “digital twin” was first used in 2010 by NASA [22]. By their definition, “*A digital twin is an integrated multi-physics, multi-scale, probabilistic simulation of a vehicle or system that uses the best available physical models, sensor updates, fleet history, etc., to mirror the life of its flying twin*”. In 2012, NASA and the US Air force introduced digital twin as the new paradigm for their vehicles [23] and in 2014, the concept of a digital twin model for manufacturing was introduced [24]. In 2017 digital twin was highlighted as one of the top 10 most promising technological trends [25]. The terminology for digital twins is now becoming standardized and is described in ISO 23247-1 as a “*fit for purpose digital representation of an observable manufacturing element with synchronization between the element and its digital representation*”. There are many benefits in digital twin implementation including, cost reduction, predicting problems, maintenance scheduling and improvement, comprehensive visibility and increasing safety [26]. An excellent example of the benefit of a digital twin in the aerospace industry is in airframe life cycle management and maintenance optimization with reduced cost and increased system reliability [27].

During the past decade, the conceptual idea for digital twin has matured and researchers have been moving forward to framework development. In the manufacturing industry, digital twin has a broad application in the product lifecycle and as a result, the framework should be refined in each section [28]. In the following, examples of digital twin framework development in human–robot collaboration and machining applications are introduced. 

In a recent study, a digital twin-driven framework was proposed for human–robot collaboration in the assembly line of a complex product [29]. The aim of this research was to combine cognitive ability of human operators with repeatability and precision of robots. For an industrial application, the assembly process of an automobile generator was used to verify the effectiveness of this method [29]. In [30], a digital representation of the human body was developed to enable ergonomic assessment during human–robot collaboration. In that study, digital human representations in digital twinning was implemented in a pick and place case study. This representation focused on work monitoring, progress prediction, etc. Considering safety standards and regulatory frameworks for close human–robot collaboration, a framework has been developed to improve efficiency of fuselage assembly in aerospace industry [31]. Results of this study showed a reduced assembly time with increased safety conditions for human–robot collaboration in a manufacturing operation. In the medical device industry, a digital twin-driven assembly approach for human–robot collaboration for the production of ventilators has been proposed in [32] and demonstrated on the assembly of alternators. Results of this research showed an improvement in assembly efficiency of over 25%, while operator workload decreased by over 20%. 

Regarding machining processes, some researchers have addressed digital twinning for the process of machine tools. Machine tool cyber twins is a term related to architectures developed for machine tools digital twin [33]. In a recent study, a multi-physics digital twin framework for a milling process was proposed [34]. This study aimed to fulfil high-fidelity, multi-scale and multi-dimensional digital twins of a workpiece during machining based on biomimicry principles. The model was assessed in monitoring and controlling the machining process of an air rudder. In another study, OPC-UA (Open Platform Communications Unified Architecture) and MTconnect were implemented as standard protocols to develop a digital twin framework for machine tools [35]. Although the proposed model only supported monitoring and data visualization of machining with a 3-axis milling machine, there is the potential for integration of more advanced artificial intelligence algorithm for optimization of machining.

Digital twin frameworks in different manufacturing environments show diversity in architecture models. These models are developed based on characteristics of specific manufacturing systems and according to the researchers understanding of the digital twin concept. Therefore, the motivation of this paper is to use available standards to develop a unified digital twin framework in manufacturing environment and then, to construct a digital twin platform for robotic drilling process as an industrial application. In digital twinning, different technologies can be implemented in different levels [28]. In the basic implementation, data are extracted from the physical space with technologies such as sensors and Internet of Things (IoT) devices. Based on the collected data, suitable models are created or updated with available technologies including data fusion, finite element models and physical models as a real-time synchronization between physical and digital twins. However, advanced technologies such as AI, blockchain, etc. are used for behaviour prediction and optimization of the system. Therefore, in this case study, visualization of drilling process parameters was carried out as the first step in digital twinning.

## 4. Digital Twin Architecture for Robotic Drilling

### 4.1. Generic Framework 

**Figure 1:**
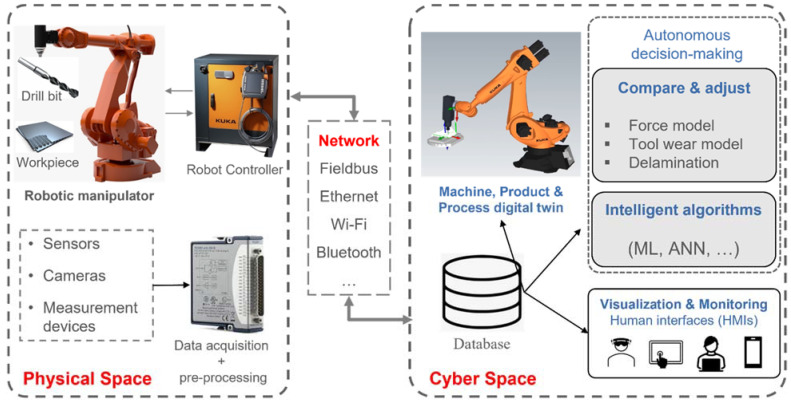
Generic framework of digital twin for robotic drilling process. ML = Machine Learning, ANN = Artificial Neural Network.

In this section, the general architecture for robotic drilling (machining) using a digital twin is introduced (see Figure 1). Information that is collected from the physical space is used for monitoring, control, optimisation and autonomous decision making [36]. There are three main elements in the digital twin: 1. Physical space, 2. Cyber space and 3. Communication network. 

**Physical space:** The physical space includes the robotic manipulator, the cutting tool and the workpiece. It also includes the robot controller, sensors, actuators and measurement devices for data collection and feedback control. Data are acquired from the physical space and send to the cyber space for analysis. The controller sends and receives control commands for robot movement. In parallel, data for the robot motion are extracted from the controller and provided to the digital twin. A drilling end-effector provides the real-time drilling process parameters such as rotational speed, feed motion, lubrication, clamping, etc. to the cyber space. To measure these processes parameters such as force, torque, temperature and acoustic emission, sensors are integrated in the drilling module. These data are collected through the data acquisition system, for data pre-processing and storage. 

**Cyber space**: In the cyber space the drilling process is analysed according to the information extracted from the robot controller and data acquisition unit. The database stores information collected from the physical space and sends it for cloud computing, visualisation, simulation, and intelligent analysis for smart decision-making. In the case of robotic drilling, the digital twin in the cyber space consists of:

- *Machine Digital Twin*: This is typically a rigid body dynamics model of the robot manipulator incorporating the end effector and drilling module. The digital twin communicates with the drilling module to record real-time process parameters such as drilling torque, speed, temperature, etc. The drill bit is explicitly incorporated within the machine digital twin.
- *Product Digital Twin*: In its simplest form this is a computer-aided design (CAD) model of the drilled component, containing information such as: number and location of drilled holes, hole diameter, component geometry, hole quality and residual stress. Some of this information is known a priori, e.g., number of holes, but some are determined either post-manufacture, e.g., hole quality, or through the process digital twin, e.g., residual stress.
- *Process Digital Twin*: The process digital twin determines the rate of material removal, hole quality, residual stress generated, tool wear and other key parameters. The process digital twin can be used in conjunction with an artificial neural network or other machine learning method for in-line prediction or smart decision making.

**Communication Network:** data flow between the physical and cyber space is the key for real time synchronization. Industrial communication networks are divided into three categories: fieldbus networks, Ethernet-based networks and wireless network [3]. Fieldbus networks (e.g., PROFIBUS, Modbus) are no longer widely used due to incompatibility with current open systems interconnection protocols. Ethernet-based networks (Profinet, EtherCat, Modbus TCP) are more common. Wireless networks provide open communication protocols for data exchange between field devices, machines and application. MTConnect is a manufacturing technical standard which provides specific information models for data exchange between CNC machine tools with predefined data structure [37] while OPC-UA covers a broader areas of application with more generic information model structure [38], and can be used for wireless communication between physical and digital twins [39]. Message Queue Telemetry Transport (MQTT) is a messaging protocol for IoT using a publish/subscribe method for information exchange.

### 4.2. Digital Twin Framework for Manufacturing Based on ISO 23247

ISO 23247 (Digital twin framework for manufacturing) provides a framework for developing a digital twin in the discrete manufacturing domain [6,40,41,42]. There are four parts to ISO 23247. ISO 23247-1 provides the general principles and requirements of a digital twin framework; ISO 23247-2 describes a digital twin reference architecture model with functional views; ISO 23247-3 provides a digital representation of manufacturing elements and ISO 23247-4 introduces different networks for information exchange. 

**Figure 2:**
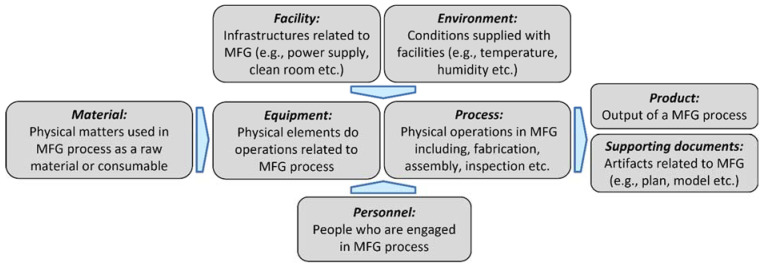
Observable Manufacturing Elements (OMEs) [6]. MFG = manufacturing.

In ISO 23247 physical entities are known as **observable manufacturing elements** (OMEs) which include personnel, equipment, material, process, facility, environment, product and supporting documents. Figure 2 presents a schematic of the OMEs related to typical manufacturing processes. The digital twin and physical twin (OMEs) are connected and synchronized by means of device communications. The digital twin can provide real-time control, off-line analytics, predictive maintenance, health check, engineering design, production control and video surveillance. This provides benefits such as in loop planning and validation, production scheduling assurance, enhanced understanding of manufacturing elements, dynamic risk management and part/process traceability [6].

**Figure 3:**
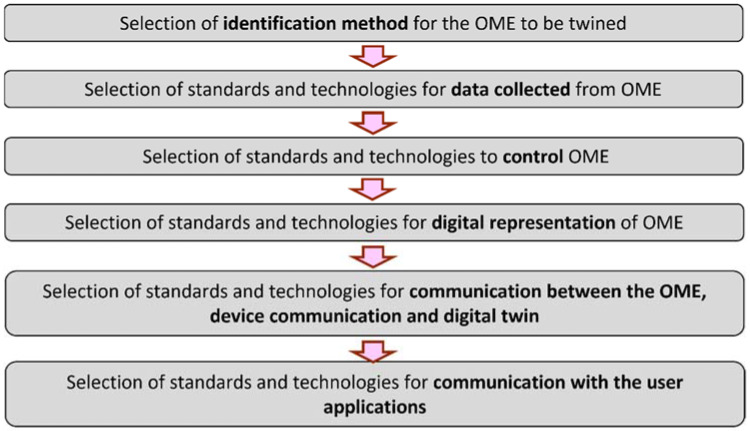
Steps to use standards and technologies to create a digital twin based on ISO 23247.

The requirements of a digital twin for manufacturing include accuracy, communication, data acquisition, data analysis, data integrity, extensibility, granularity, identification, management, product life cycle, security, simulation, synchronization, viewpoint and hierarchical modelling of digital twin for manufacturing [6]. To apply this framework to a manufacturing operation, different standards and technologies are used in the different steps, as outlined in Figure 3.

**Figure 4:**
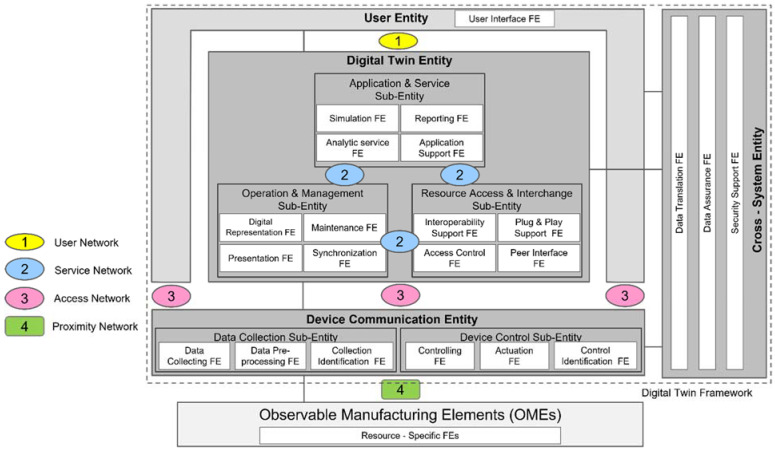
Functional view of entity based digital twin reference architecture combined with Network view. Adapted from [40,42]. OME = observable manufacturing element; FE = functional entity.

In ISO 23247-2, the digital twin reference architecture is introduced in terms of **domains**, where different tasks are performed and **entities**, where domains are divided into system levels and subsystem levels [40]. A domain consists of one or more OMEs in the physical space. Sub-entities may also be considered in the domain. To understand the functionalities of each sub-entity, **functional entities** (FEs) are introduced, implementing an entity-based reference model. As illustrated in Figure 4, the device communication entity, the digital twin entity, and the cross-system entity consist of FEs that define the purpose of each sub-entity. In the device communication entity, the data collection sub-entity functions as data collection, data pre-processing and collection identification. 

To construct a digital twin, a digital representation of the OMEs is needed in terms of static and dynamic information attributes. Static information remains the same during the process while dynamic information changes throughout the process. Different standards exist depending on the objective of the digital twin [41]. Examples include:

- ISO 10303-238: Product data representation and exchange, model based integrated manufacturing [43]
- ISO 10303-242: Product data representation and exchange, managed model-based 3D engineering [44]
- ISO 13399: Cutting tool data representation and exchange [45]
- ISO 23952: Quality information framework, and integrated model for manufacturing quality information [46]
- MTConnect: Protocol for exchange of information between shop floor devices [37]
- IEC 62541 (OPC-UA): Protocol for machine to machine information exchange [47]

Another important factor in the architecture model is information exchange between entities (see Figure 4). Four networks are considered for data exchange: (1) the user network between the user entity and the digital twin entity; (2) the service network for data exchange between sub entities in the digital twin entity; (3) the access network that connects the user entity and the digital twin entity with the device communication entity and (4) the proximity network for data exchange between OMEs and the device communication entity [42].

Through the user network, the user entity uses the digital twin models for higher level actions. The user entity can connect to the digital twin over the internet using hypertext transfer protocol and representational state transfer. Graphical information can be shared using WebGL and Open GL. A web-based cloud or database can be used for data sharing.

In the access network, data collected from the OMEs are transmitted to the digital twin entity while controlling commands are transmitted from the user entity or digital twin entity to the device control sub entity, using MTConnect, OPC-UA or MQTT. In the proximity network, the device communication entity transmits control commands to the OMEs and collects data from the OMEs by means of sensors. Available options for proximity network are industrial Ethernet (e.g., Profinet, EtherCAT, Modbus …), wireless and proprietary networks. 

## 5. Digital Twin Architecture for ISO 23247: A Case Study of Robotic Drilling

**Figure 5:**
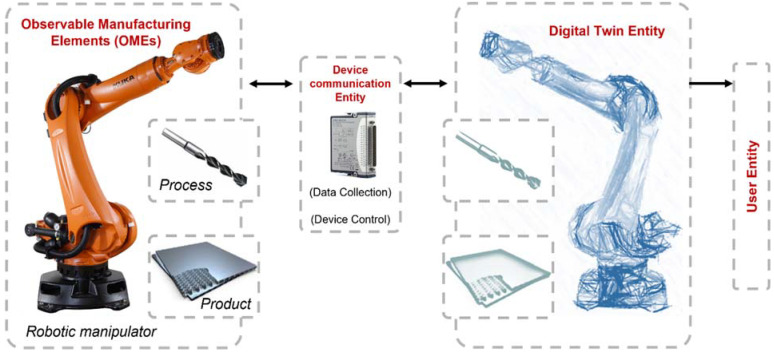
Schematic diagram of digital twin framework for robotic drilling processes.

Figure 5 presents a schematic diagram of the digital twin framework for robotic drilling based on ISO 23247. The digital twin entity shown on the right of Figure 5 is used as the digital representation of the physical OMEs, shown on the left of Figure 5, including the robotic manipulator, the drilling process and the product.

***OMEs:***

 *1*- *Equipment* *:*

**Figure 6:**
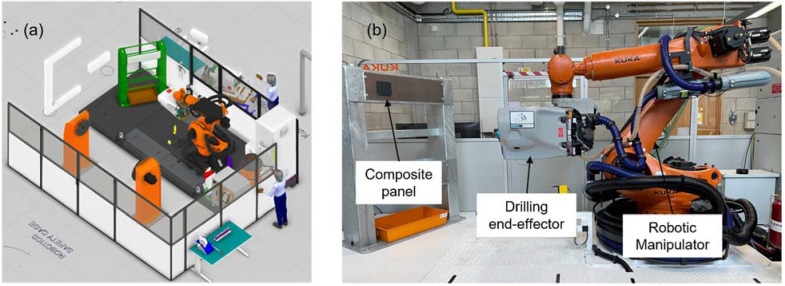
Manufacturing cell for robotic drilling (**a**) 3D CAD model, (**b**) physical space at University of Limerick, Confirm Smart Manufacturing laboratory.

Figure 6 shows the manufacturing cell for a robotic drilling process at the University of Limerick, including a KUKA robot KR210 R3100 Ultra with a KRC4 controller. KR210 R3100 Ultra is a six-axis industrial robot with a reach of 3095 mm and maximum payload of 210 kg. It has a Kuka Aerospace multifunction end-effector with a drilling and vision module (COGNEX 5400 camera), main control cabinet, a data processing pilot to perform work sequences and a Profinet fieldbus system to acquire information from sensors and transfer data. V7000 is the data processing pilot software, developed by Kuka Systems Aerospace (Le Haillan, France) to control the drilling operation sequences in a primary/replica manner. In the multifunction end-effector, different modules (e.g., drilling, sealant installation, vision and fastener) are available. To satisfy hole positioning accuracy and perpendicularity, the end effector has normality and slippage compensation function. This capability enables precise adjustment of the drilling axis perpendicular to the panel and at the same time prevents the robot from slipping due to the high clamping force. Hole positioning accuracy of vision system is +/−0.1 mm with repeatability of +/−0.25 mm.

By applying both global and local relocation procedures, the vision system defines the base frame for the geometric points downloaded to the robot arm and resets the coordinates of the holes based on reference points. The relocation operation makes it possible to adjust the robot trajectories according to the part geometry.

 *2*- *Materials* *:*

The materials suitable for the robotic drilling system include aerospace and automotive grade alloys including advanced, high-strength aluminium and titanium-based alloys, and carbon-fibre reinforced polymers (CFRP).

 *3*- *Product* *:*

The Kuka Aerospace multifunctional end-effector is used in the aerospace industry for drilling, riveting and sealing operation on fuselages. At the University of Limerick it is used for drilling of coupon-sized products, as well as larger composite structures prone to vibration and flexing during the drilling and fastening process.

 *4*- *Process* *:*

Once the workpiece material is identified, a suitable drill bit is selected for the targeted hole size, the coordinates of holes are determined, and the process parameters and drilling sequence are specified. The robotic manipulator is used for spatial positioning of the tooling system while the drilling end-effector carries out the drilling process. The coordinates of the holes are supplied to the robot manipulator for positioning and the drilling process parameters are supplied to the end effector. During drilling, data are collected from sensors integrated into the end effector, the robotic cell and the controller of the robot arm. There is a dedicated controller for the robotic manipulator, while there is a second controller that controls the remaining cell functions, such as door interlocks. Data collected are transmitted to the cell controller through fieldbus and are used for digital twin creation and analysis. 

## 6. Experimental Procedure for Robotic Drilling

**Figure 7:**
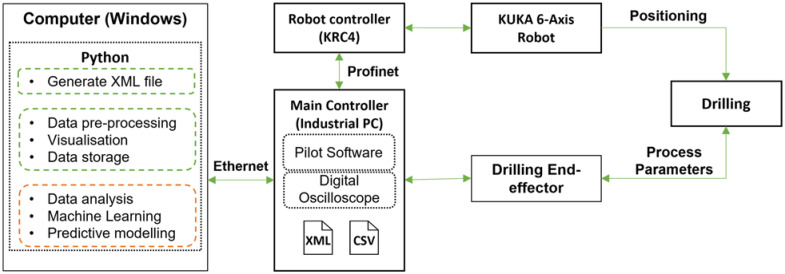
Process flow for robotic drilling process execution and monitoring.

The main objective of the experiment is to demonstrate real-time visualisation of the drilling feed and rotation torque as a first step in digital twinning. Figure 7 demonstrates the process flow for executing the drilling process using an industrial robot and real-time visualisation of the process parameters. The main controller (Industrial PC) is connected directly to the drilling end-effector and the robot controller. During drilling, the digital oscilloscope located in the main controller, displays real-time sensor data at a sample rate of 250 Hz. An external Windows laptop is connected to the main controller via Ethernet cable to allow real-time data communication. Prior to the drilling operation, an XML (Extensible Markup Language) file is generated via Python (Windows laptop), which includes the reference and drilling points, the robot trajectory and the drilling process parameters. This XML file is then uploaded to the pilot software (V7000 in the industrial PC) to perform the drilling process. 

A vision module in the end-effector recognises the location of the reference points and calculates the coordinates of drilling points as identified in the XML file. Subsequently, the drilling operation occurs through the feed and rotation motor. In this experiment, the workpiece material is a 4 mm thick CFRP panel, while a 4.8 mm diameter diamond-like carbon coated Diager drill bit with a 135° point angle is used. For the drilling operation, with a feed speed of 250 mm/min was used, while the rotation speed of the drill bit was set at 2000 rpm.

**Figure 8:**
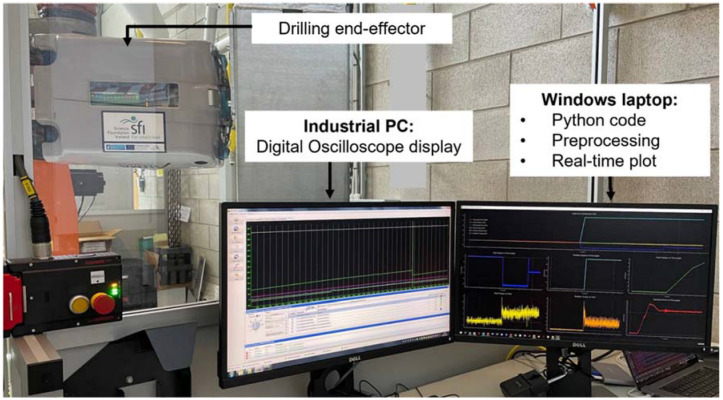
Experiment equipment for real-time data collection and visualization of robotic drilling process.

Figure 8 shows the equipment for data collection and visualization of the robotic drilling process. From the figure, the drilling end-effector is executing the drilling operation, the industrial PC (digital oscilloscope) extracts the data from sensors embedded in the end-effector and the Windows laptop demonstrates the real-time drilling process parameters.

**Figure 9:**
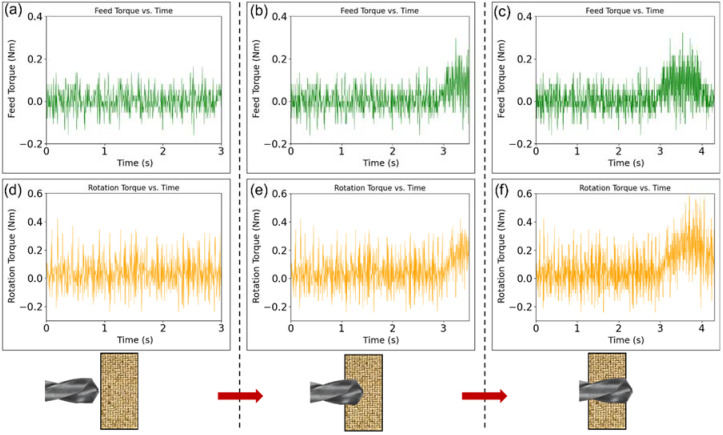
Real-time data plots of feed torque (**a**–**c**) and rotation torque (**d**–**f**) in different drilling steps extracted from servo motors located in the end-effector causing drilling feed and rotation motion, respectively.

The data are pre-processed and displayed using Python. The PyQtGraph library displays the data at the same sample rate as the digital oscilloscope to allow real-time visualisation. These data can be used to create a predictive model to enhance the drilling process or for further analysis and optimization through machine learning. Figure 9 displays changes in rotation and feed torque for a single hole drilling. These data are extracted from servo motors located in the end-effector to provide rotation and feed motion to the drill bit. In pre-possessing, the effect of energy loss from the power transmission chain (i.e., pullies, belts, ball screw, etc.) was eliminated by subtracting torque values from air cutting. Before the drill bit engages with the workpiece (Figure 9a,d), torque values are almost zero. Feed and rotation reach their maximum values in full engagement (Figure 9b,e) and then decrease when the drill bit exits the workpiece (Figure 9c,f). These recorded data can be used to establish the stable drilling status and track any deviation from the stable condition which may be caused by tool wear or breakage, overheating, excessive workpiece deformation, etc. The data can also be used to develop data-driven models for prediction of tool failure or poor-quality holes. 

Other OMEs which can be considered for this system include:

1. *Personnel*: Operators, engineers, maintenance staff, research & development staff.
2. *Facilities*: Ventilation, heating, air pump, illumination, vacuum pump, oil/lubrication pump.
3. *Environment*: Temperature, luminance, humidity.
4. *Supporting documents*: Drilling process plan, CAD/CAM files, process measured values, quality control.

## 7. Device Communication Entity 

**Figure 10:**
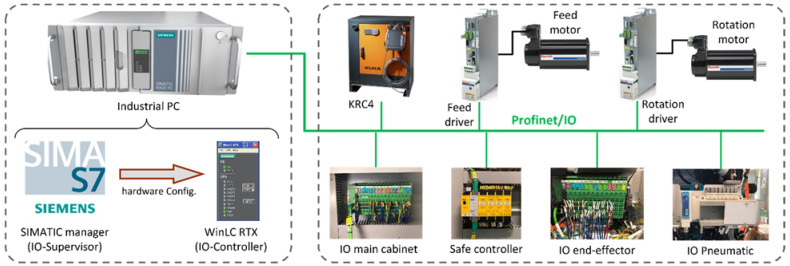
Profinet/IO as proximity network of the robotic cell. The industrial PC hosts IO-Supervisor and IO-Controller while IO-Devices are connected to industrial PC through Ethernet cable.

The proximity network used is Profinet/IO. Key elements of a Profinet/IO system are the input and output (IO)-Controller that controls the automation system, IO-Devices that are controlled by the IO-Controller and the IO-Supervisor software to define hardware configuration and parameter settings. Here, the IO-Supervisor is SIMANTIC manager and the IO-Controller is a PC-based PLC controller. Figure 10 shows the Profinet/IO fieldbus system. From the figure, the industrial PC is the control unit of the robotic cell, which collects data and sends control commands to the OMEs. The KRC4 controller reads commands from the main controller (Industrial PC) and sends requested information to the control unit. Feed and rotation motors in the end-effector receive signals from the respective drivers. Other devices are the IO main cabinet, the safe controller, the IO end-effector and the IO pneumatic. These are used to send and receive data from sensors in the robotic cell and end-effector.

## 8. Advantages of Proposed Framework

Using the ISO 23247 architecture model, simulation, analysis and visualisation of OMEs can be performed. For example, virtual commissioning of robotic drilling enables a detailed overview of the process, problem detection, optimisation of robot trajectory and failure prevention. For real-time visualisation of the robotic arm, information obtained from the robot controller (e.g., OPC-UA server/client communication) can drive a 3D model of the robot manipulator and study multibody dynamic behaviour of the manipulator during drilling. Drilling process parameters such as rotation speed, feed rate and thrust force can be monitored for an improved understanding of the process. 

## 9. Conclusions

Based on ISO 23247 a digital twin framework for an advanced robotic drilling system has been proposed. Three entities have been defined: the device communication entity, the digital twin entity and the user entity. The relevant OMEs for robotic drilling include the industrial robot and end-effector (equipment), aluminium, titanium or composite (material), aircraft fuselage (product) and drilling (process). In the case of the device communication entity, OPC-UA protocol has been identified as the information model to monitor robot axis motion. Such a digital twin framework for robotised drilling may be used for virtual commissioning of robotics, or real-time 3D visual representations. There are some limitations regarding this work however. The development of a comprehensive digital twin that covers all aspects of robotic drilling process is costly and time consuming. Future work will build on the proposed digital twin framework, through the development of force models, tool wear prediction, etc. Artificial intelligence algorithms will be implemented to predict system behaviour and optimise process efficiency.
